# Teledentistry: A Boon Amidst COVID-19 Lockdown—A Narrative Review

**DOI:** 10.1155/2021/8859746

**Published:** 2021-02-16

**Authors:** Shantanu Deshpande, Devendra Patil, Amol Dhokar, Parin Bhanushali, Farhin Katge

**Affiliations:** ^1^Department of Pediatric and Preventive Dentistry, TPCT's Terna Dental College, Navi Mumbai, India; ^2^Department of Oral Medicine, Diagnosis and Radiology, TPCT's Terna Dental College, Navi Mumbai, India

## Abstract

The recent spread of severe acute respiratory syndrome coronavirus 2 (SARS-CoV-2) and its associated coronavirus disease (COVID-19) has caused widespread public health concerns. Despite huge efforts to contain the disease spread, it is still on the rise because of the community spread pattern of this infection. In order to prevent the community spread, a nationwide lockdown was implemented, due to which many restrictions were imposed on movements of citizens within the country. Since the dental professionals were at the forefront of acquiring the infection, the majority of the dental clinics were shut for routine dental procedures. Only emergency treatment was provided to the patients. However, due to restrictions in movement, it was difficult for the patients to visit the clinics for routine check-ups. This was overcome by the advancements in technology which has a major impact on medicine. Due to increased usage of smartphones and related software applications, the clinical data exchange was facilitated between patients and clinicians which has been termed as “teledentistry.” Teledentistry is a combination of telecommunications and dentistry, involving the exchange of clinical information and images for dental consultation and treatment planning. This technology served as a boon for the dentists to manage dental emergencies during the lockdown period. This narrative review discusses teledentistry and its applications in general and specialty dental practice amidst the COVID-19 lockdown.

## 1. Introduction

An unusual type of pneumonia appeared in Wuhan, China, in December 2019. The etiological factor was found to be coronavirus, which was renamed by the World Health Organization (WHO) as “Coronavirus disease 2019” (COVID-19) [1]. A rapid spread of COVID-19 occurred across both China and the world, due to which the WHO declared the coronavirus disease as a pandemic on March 11, 2020 [2]. In view of the pandemic, the government of India declared a nationwide lockdown on March 24, 2020, with restrictions on travel and social gatherings.

It was recommended to avoid visits to the hospitals, dental setups, or other medical facilities as it serves as a source of cross infection [3]. The dental setting proved to be a significant source of transmission as most of the dental procedures involve the production of aerosols and droplets contaminated by microorganisms [4]. This makes the dentist prone to get infected from patients and spread the infection to their families. This developed a sense of fear and anxiety in the minds of the dentists [5]. To overcome this, new models of consultation need to be encouraged to provide minimal contact between dentists and patients.

In today's time, with high-speed mobile data, the internet is playing an important role in building strong communication between dentists and patients. With the usage of smartphones, laptops, and various video conferencing software applications, it is possible to perform “teledentistry,” a new concept to render dental care across distance [6]. It has completely modified the traditional approach of working as it promotes a virtual method of consultations and follow-up instead of face-to-face clinical evaluation. This has proved extremely beneficial in times of the COVID-19 lockdown, minimizing patient visits to the dental clinic unless absolutely essential. The purpose of this narrative review is to describe the importance of teledentistry for general and specialized dentists amidst lockdown to manage dental emergencies of patients.

## 2. What Is Teledentistry?

Teledentistry is a combination of telecommunications and dentistry, involving the exchange of clinical information and images over remote distances for dental consultation and treatment planning [7]. The term “teledentistry” was first used in 1997, when Cook defined it as “the practice of using video-conferencing technologies to diagnose and provide advice about treatment over a distance” [8].

## 3. History of Teledentistry

Teledentistry, similar to telemedicine, was first used by NASA in the 1970s and then by the US military [9]. The initial concept of teledentistry was developed as part of the blueprint for dental informatics in 1989 [10]. The US army's Total Dental Access (TDA) project which began in 1994 was the frontier of teledentistry that enabled the dentists from the US armed forces to have a specialist consultation at a medical center regarding their patients [11]. As technology has advanced, new opportunities for teledentistry have been created. Technologies currently available are leading to a change in the dynamics of dental care delivery.

## 4. How Does It Work?

Technological innovations and high-speed internet networks can be applied in the field of medicine and dentistry. In this pandemic, teledentistry can prove to be of great help for patients with dental emergencies. It can happen in two forms:

- Real-time consultation
- Store and forward

**Figure 1:**
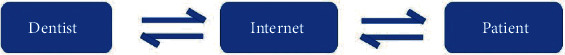
Real-time consultation.

Real-time consultation (Figure 1) involves a video conference in which dental professionals and the patient, at different locations, may see, hear, and communicate with one another using advanced telecommunication devices and high-speed internet connections [12, 13].

**Figure 2:**
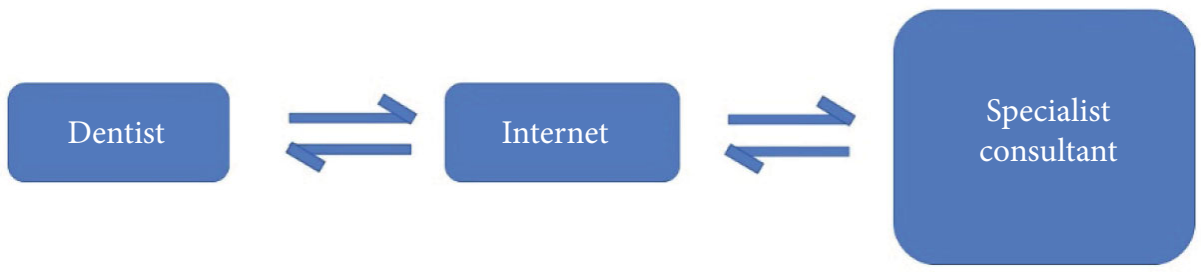
Store-and-forward method of consultation.

Store and forward (Figure 2), on the other hand, involves the exchange of clinical information and static images collected and stored in the telecommunication equipment. The dentist collects all the required clinical and radiographic information from the patient. This information is then sent to the specialist for consultation and treatment planning. The treatment is thus provided in a far more timely, targeted, and cost-effective manner [13].

## 5. Scope of Teledentistry

Teledentistry has the ability to improve access and delivery and lower the cost of oral healthcare eliminating disparities between the rural and urban communities [14, 15]. It helps people to receive specialized healthcare measures in remote parts of the world due to advancements in the field of telecommunication [16]. Lienert et al. found that telemedical services were helpful for dental trauma cases in a Swiss telemedical center and provided valuable support in the absence of a specialty dentist [17].

The use of teledentistry for specialist consultations provides an aid for diagnosis, treatment planning, and coordination, by sharing clinical and radiological photographs of the patient among dentists [18]. It also aids in obtaining second opinions, preauthorization, and other insurance requirements instantaneously, using real images of dental problems instead of tooth charts and written descriptions [19]. Teledentistry also supplements traditional teaching methods in dental education, providing new opportunities for dental students and dentists [20].

## 6. Armamentarium Involved in Teledentistry

For most dental applications, store-and-forward technology provides excellent results without excessive costs for equipment or connectivity. A typical store-and-forward teledentistry system consists of a computer, an intraoral video camera, and a digital camera for the pictures. It also consists of a modem and an internet connection [13, 19, 21]. Nowadays, due to advancements in telecommunications, smartphones with integrated video conferencing applications are used for teleconsultations between the dentist and the patient.

## 7. Applications of Teledentistry in Lockdown

The transmission routes, treatments, and outcomes of COVID-19 have been extensively researched upon. It is clear for now that the mode of transmission is through contact in the form of droplets [22]. The Occupational Safety and Health Administration (OSHA) has placed dental healthcare professionals in a very high exposure risk category stating that dentists work in close proximity to the patient's oral cavity [23]. The literature shows that many dental procedures are aerosol producing, having the potential to spread infections to dental personnel and other people in the dental office [4].

Online conversations allow the exchange of several types of data like written or voice messages for diagnostic doubts as well as therapeutic suggestions, video messages for a better evaluation of a patient's requirements, and descriptions of problems in his own words [24]. Surely, high-quality images are the most common means of communication in teledentistry, showing clinical examination reports, radiological investigation reports, or simple photos of lesions [25]. Remote consultations can be performed either among dentists or between dentists and patients.

## 8. Teledentistry in General Dental Practice

The general dentist serves to be the first point of contact for a patient in times of emergency situations. The most common dental emergencies faced by a general dentist during lockdown are pain, swelling (intraoral or extraoral), or both. In order to handle these emergency situations, the first line of treatment involves the prescription of suitable antibiotics and analgesics. If these symptoms do not subside, the dentist may advise the patient to visit the clinic for an emergency procedure.

In cases of dislodged temporary cement, the patient will be asked to keep the cavity clean with normal tooth brushing post every meal. If the tooth is treated endodontically, then the patient may be asked to rinse the tooth with water diluted with hydrogen peroxide using a syringe without a needle [26]. A sterilized cotton pellet is to be kept in the cavity before meals. Hot and cold foodstuffs are to be avoided by such patients, and they are asked to chew from the opposite side. The dentist tries to solve as many problems as possible with the help of teledentistry. However, in case of certain specific emergencies, he has to consider a consultation with a specialist dentist.

## 9. Teledentistry in Specialty Dental Practice

### 9.1. Role in Oral Medicine and Diagnosis

The majority of the patients with oral ulcerative lesions or soft tissue ulcers can get their photographs clicked with smartphones and send them to the dentist. The dentist can analyze them and give suitable medications through teleprescription after enquiring about the proper medical and allergy history of the patient. Digital Orthopantomogram (OPG) and Cone Beam Computed Tomography (CBCT) reports can be sent to oral and maxillofacial radiologists in cases of cysts or tumors for prompt diagnosis, following which a due course of treatment can be planned. Torres-Pereira et al. [27] suggested that distant diagnosis is an effective alternative in the diagnosis of oral lesions using the transmission of digital images by email.

### 9.2. Role in Oral and Maxillofacial Surgery

In the case of oral and maxillofacial surgery, the most common complaints arising are of pain in the third molars. Such patients can send clinical and radiographic images to dentists which can be forwarded to a specialist for a consultation. Due to the COVID-19 pandemic, the incidence of dental trauma and nonurgent treatments has decreased [28]. Duka et al. [29] showed that the clinical diagnosis of impacted or semi-impacted third molars assisted by the telemedicine approach was equal to the real-time assessment of clinical diagnosis. Furthermore, as reported by Saad Ahmed and Omar [30], oral surgery benefits from teledentistry, not only in terms of dental procedures but also in terms of monitoring postoperative conditions of the patients.

### 9.3. Role in Endodontics

The most common complaint of the patients in lockdown is dental pain and swelling. In such cases, they can have a telephonic conversation with the dentists informing them about the symptoms experienced by them. Ather et al. [31] categorized endodontic interventions into primary and secondary treatment protocols. For certain specific cases like symptomatic reversible pulpitis, analgesics can be prescribed. If proven ineffective, a pulpotomy procedure can be performed as a secondary protocol. The dentist can then prescribe them a suitable course of antibiotics or take a clinical judgment if the case requires immediate clinical intervention depending upon its severity. Zivkovic et al. [32] demonstrated that teledentistry based on the internet as a telecommunication medium can be successfully utilized in the diagnosis of periapical lesions.

### 9.4. Role in Orthodontics

Orthodontic emergencies include breakage of brackets and loosening of wire. Such complaints can easily be handled by the dentist over the telephone, and the patient can be instructed how to temporarily fix the problem. According to Berndt et al. [33], interceptive orthodontic treatments were provided by sufficiently trained general dentists and supervised remotely by orthodontic specialists through teledentistry. This proved to be a viable approach to reduce the severity of malocclusions in disadvantaged children when referral to an orthodontist is not feasible. Cook et al. [34] tested an online teledentistry service and showed that it helped to reduce the high level of inappropriate orthodontic referrals to consultants and provided general dental practitioners with quick access to advice that would enable them to tackle a wider range of cases themselves.

### 9.5. Role in Prosthodontics

In the field of prosthodontics, the most common complaints involve dislodged prosthesis and breakage in dentures. Such complaints can be handled by dentists by guiding the people on how to manage the situation at home by speaking to laboratory technicians to collect the dentures from the patient's residence and temporarily fixing them.

### 9.6. Role in Periodontics

Teledentistry in the field of periodontics makes use of the store-and-forward method. The dentist can collect all photographs of patients (intraoral and extraoral) along with required radiographs and send them to the periodontist for consultation. The periodontist can then view those pictures and radiographs to determine a suitable treatment plan. Then, the dentist and periodontist can decide whether to treat the patient on an emergency basis, or the treatment can be postponed till the lockdown is lifted. The web-based teledentistry consultation system developed for the US Department of Defense dental clinics showed that referrals to oral surgery, prosthodontics, and periodontics had the highest number of consults [35]. Fifteen patients underwent periodontal surgery at Fort Gordon, Georgia, and a week later, their sutures were removed at a location 150 miles away under the telesupervision of the periodontist. Only 1 patient made the return trip for a follow-up procedure.

### 9.7. Role in Pediatric and Preventive Dentistry

Children are more susceptible to acquire infection than adults. Hence, the extra visits of children to the dental clinics can be avoided using teledentistry. The parents can send intraoral pictures of the children along with the pain history to the pediatric dentist. The pediatric dentist can then evaluate the severity and determine a suitable treatment plan for the child. The parents can be guided to perform adequate home care measures to prevent further decay and ensure meticulous oral hygiene. The parents can also be guided in cases of dental trauma to their children at home. Emergency ice packs should be applied to the site of trauma. If the tooth has fractured or completely avulsed, the parents can be asked to store the tooth in milk. The patients are then called to the clinic for either reimplantation in cases of complete avulsion or a biological restoration in cases of a fractured tooth. Kopycka-Kedzierawski et al. [36] demonstrated that the intraoral camera is a feasible and potentially cost-effective alternative to a visual oral examination for caries screening, especially early childhood caries, in preschool children attending childcare centers.

## 10. Advantages of Teledentistry in COVID-19 Lockdown

### 10.1. Management of Preliminary Emergencies

The dentist can manage primary emergencies by prescribing suitable antibiotic therapy and prescribing home care measures to the patient. This would help in prolonging the treatment until the lockdown is lifted.

### 10.2. Aiding in Specialist Consultations

Teledentistry serves as an efficient medium for having a specialist consultation without the need to visit a dentist during the lockdown. This facilitates a more efficient and immediate formation of a treatment plan for the patient.

### 10.3. Follow-Up Visits Can Be Avoided

The dentist after performing emergency procedures like extractions or emergency access opening in cases of swelling can obtain a proper follow-up for the patients with the help of photographs clicked by the patient of the site of infection.

## 11. Pitfalls of Teledentistry

Even though in these testing times teledentistry serves as a boon for dentists, nothing can match the accuracy of the diagnosis of the patient performed clinically. In teledentistry, the various crucial steps of diagnosis cannot be performed, palpation and percussion being the most important ones.

### 11.1. Treatment Requires Visits to the Clinic

Teledentistry helps only in the preventive and diagnostic procedures. If a patient requires treatment, he has to visit the clinic for procedures like restorations, endodontic treatments, and extractions.

### 11.2. Virtual Examination

Diagnosis is based on clinical photography that may change on face-to-face communication [37]. The accurate display on intraoral photographs or video recordings may be different from what is present actually. Additional diagnostic aids such as percussion and palpation cannot be performed.

## 12. Informed Consent in Teledentistry

Concerns about the confidentiality of dental information arise from the transfer of medical histories and records as well as from general security issues of electronic information stored in computers [38]. Utmost care should be taken to ensure that the privacy of the patient is not compromised. However, the dentist should also make the patient aware that during the electronic exchange of information, there are chances of possible interception even if maximum security measures are implemented [13]. Proper informed consent should be taken from the patient. The patient should also be informed of the inherent risk of improper diagnosis or treatment due to the failure of the technology involved [14].

The medicolegal and copyright issues also have to be considered in teledentistry practice [39]. Such problems arise primarily due to a lack of well-defined standards [14]. There is no method to ensure the quality, safety, efficiency, or effectiveness of information or its exchange. There are privacy and security issues along with remuneration, fiscal, and taxation issues associated with electronic commerce. Many of the legal issues, such as licensure, jurisdiction, and malpractice, have not yet been definitively decided by legislative or judicial branches of various governments [40].

## 13. Conclusion

Even with high standards of knowledge and practices, dental practitioners around the globe are living in a state of anxiety due to the pandemic. Teledentistry in times of this lockdown has proved to be a boon for dentists and dental specialists in rendering efficient oral healthcare to their patients. Whether it is video conferencing for performing diagnosis or giving home care measures to patients, it has enabled dentists to solve many oral health-related problems virtually. Although it is not error-free and involves many medicolegal issues, in the testing times of COVID-19, teledentistry has proven to be extremely beneficial for handling emergency situations as far as possible, without causing the patients to visit the dental clinic unless absolutely essential.

## Data Availability

The data would be available on request to the corresponding author.
